# Solid Additives for Spontaneously Spreading‐Processed Organic Photovoltaics

**DOI:** 10.1002/advs.202512384

**Published:** 2025-08-26

**Authors:** Huitong Deng, Qianqing Jiang, Dianyi Liu

**Affiliations:** ^1^ Zhejiang University Hangzhou Zhejiang 310027 China; ^2^ Zhejiang Key Laboratory of 3D Micro/Nano Fabrication and Characterization Research Center for Industries of the Future Department of Electronic and Information Engineering School of Engineering Westlake University Hangzhou Zhejiang 310030 China; ^3^ Westlake Institute for Optoelectronics Hangzhou Zhejiang 311421 China; ^4^ Division of Solar Energy Conversion and Catalysis at Westlake University Zhejiang Baima Lake Laboratory Co., Ltd. Hangzhou Zhejiang 310000 China; ^5^ Westlake Optoelectronic Technology Co., Ltd. Hangzhou Zhejiang 310024 China

**Keywords:** green solvents, organic photovoltaics, solid additives, spontaneous spreading

## Abstract

The spontaneous spreading (SS) process offers a unique solution‐processed platform for fabricating organic photovoltaics (OPVs) under open‐air conditions. Yet its development is hindered by limited strategies to achieve uniform film thickness and homogeneous phase morphology. In this work, an SS process is developed using the green solvent o‐xylene and mediated by solid additives to regulate interfacial tension, enabling the formation of uniform SS‐films on the water surface. The aromatic solid additive phenanthrene (PAT) extended the drying time of the wet film, effectively suppressed excessive acceptor aggregation, enhanced molecular packing order, and optimized phase‐separated morphology for the SS‐film. The OPV device with SS‐film as the active layer achieved a champion efficiency of 16.4%. To the best of current knowledge, this is the first attempt to introduce solid additives in SS‐processed OPVs, offering a versatile strategy to simultaneously adjust film‐forming kinetics and phase morphology. This study presents a simple approach to fabricating solution OPVs with solution processability and reproducibility.

## Graphical abstract

This work developed a spontaneous spreading process with solid additives for the fabrication of organic photovoltaics (OPVs). By regulating interfacial tension, the solid additives enable the formation of uniform films on the water surface. A remarkable power conversion efficiency of 16.4% is recorded in the OPVs using phenanthrene (PAT) as the additive.

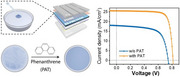

## 1 Introduction

The spontaneous spreading (SS) process offers a simple and efficient method for preparing organic photovoltaics (OPVs).^[^ ^1^, ^2^, ^3^ ^]^ This technique utilizes Marangoni Flow to achieve uniform liquid film coverage, providing a fast and energy‐efficient way for fabricating the active layer.^[^ ^1^ ^]^ Compared to traditional methods like spin‐coating, it significantly reduces material waste and energy consumption.^[^ ^4^ ^]^ However, the inappropriate interfacial tension often leads to non‐uniform thickness and poor molecular arrangement of the SS‐film, which hinders the development of high‐performance SS‐processed OPVs.

Additive engineering enables precise control over the bulk‐heterojunction morphology and phase separation size of the active layer by the spin‐coating method, which is crucial for the development of OPV.^[^ ^5^, ^6^, ^7^ ^]^ Several works have also shown that the additive engineering has become a key strategy to adjust surface tension and optimize the morphology of the active layer by the SS process.^[^ ^1^, ^3^, ^8^, ^9^, ^10^, ^11^, ^12^ ^]^ The SS‐processed PM6 film based on chlorobenzene solvent was transferred onto the acceptor layer to construct a planar heterojunction, where an addition of 1,8‐diiodooctane (DIO) effectively regulated PM6 orientation, yielding an optimized device with a power conversion efficiency (PCE) of 7.36%.^[^ ^12^ ^]^ Furthermore, a low ratio of silicone oil (0.02 vol%) could substantially reduce the surface tension of the active layer in the green solvent o‐xylene, and the device showed a champion PCE of 17.4%.^[^ ^13^ ^]^ More recently, a novel strategy introduced amphiphilic additives in chloroform‐based PM6:Y6 to form a uniform film in ultrafast (≈1 s) time and achieved a champion PCE of 15.2% in SS‐OPV.^[^ ^14^ ^]^ These studies have established effective methods in specific systems by introducing trace amounts of solvent additives.

Besides the liquid additives, solid additives offer superior advantages in spin‐coating processed OPVs, including good morphology control ability, ease of post‐treatment, and enhanced device stability.^[^ ^15^, ^16^, ^17^, ^18^, ^19^ ^]^ Among them, aromatic solid additives have a higher affinity for the conjugated backbones of active layer materials, tending to increase their miscibility with fullerene.^[^ ^5^ ^]^ However, their simultaneous modulation of surface tension and crystallization kinetics remains underexplored in the fabrication of SS‐processed OPVs.

In this work, we introduce solid additives into the active layer precursor solutions with green solvent o‐xylene to reduce the solution surface energy and promote the uniformity of the active layer prepared with the SS process. Aromatic solid additive molecules with different substituents, conjugated structures, or symmetries, which are commonly used in the spin‐coating process, are evaluated in the study. With the addition of solid additives, the solution surface tension can be dramatically reduced, facilitating the spreading of the active layer blend solution on the water surface. The OPV device with SS active film using phenanthrene (PAT) as the additive achieved a PCE of up to 16.4%. These results demonstrate that the solid additives play a crucial role in guiding future SS process optimization and enhancing device performance.

## 2 Results and Discussion

**Figure 1:**
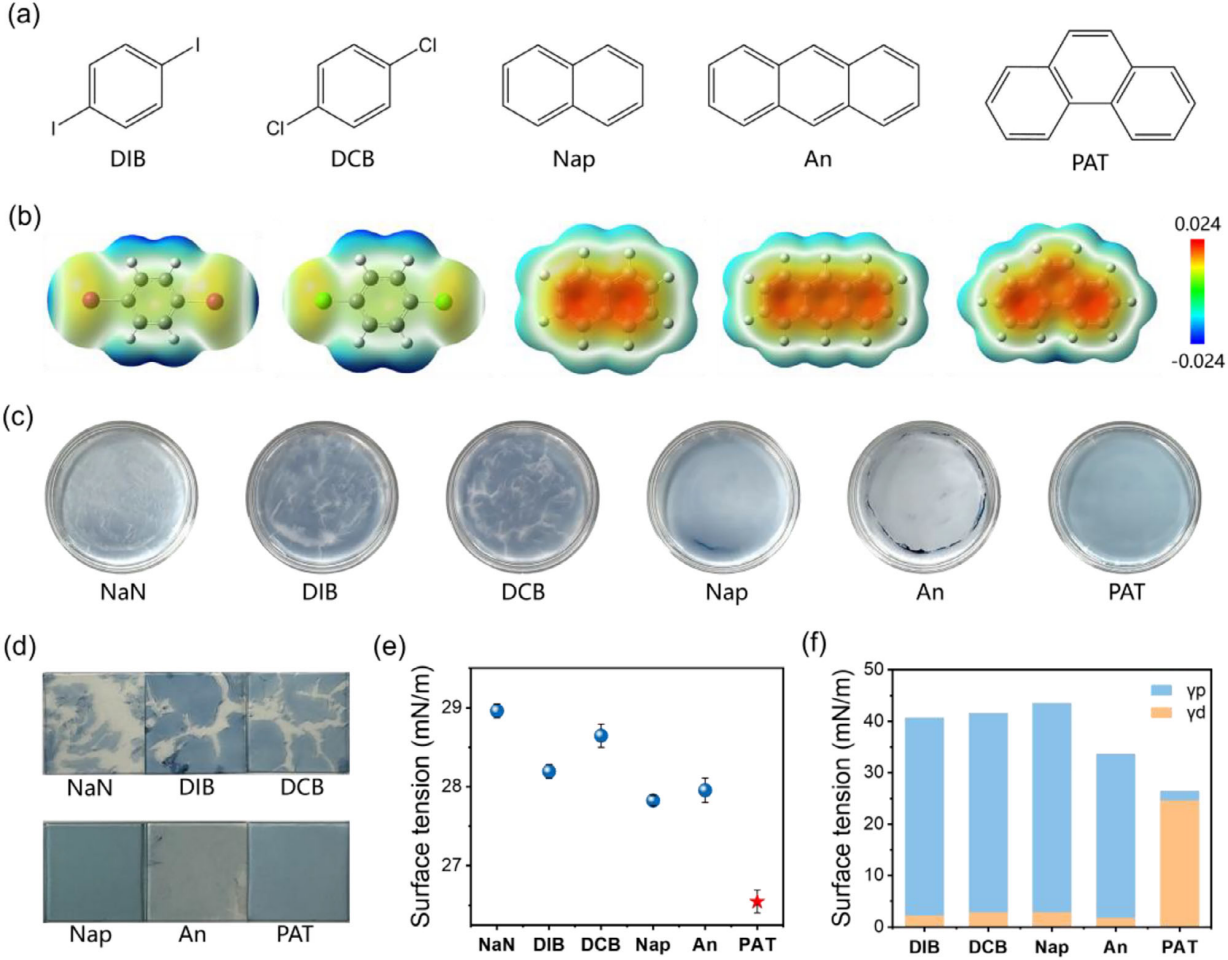
Properties of solid additives. a) Chemical structures and b) ESP patterns of different solid additives. c) Photographs of SS‐PM6:Y6 films with different solid additives after spreading on the water surface. (The diameter of the petri dish is 65 mm.) d) Photographs of transferred SS‐PM6:Y6 films. (The length of the square substrate is 15 mm.) e) Surface tension of the solid additive solutions in o‐xylene. f) Surface tension of the solid additive films, including dispersion (γd) and polarity (γp).

In contrast to non‐aromatic additives, aromatic solid additives have similar properties to the host solvent and good miscibility with π‐conjugated polymers and fullerene molecules.^[^ ^20^ ^]^ Table S1 (Supporting Information) summarizes recent literature demonstrating the use of aromatic solid additives to modulate film morphology and enhance device performance in the spin‐coating process. In our work, we selected aromatic solid additives with conjugated ring structures to investigate the effects on the spontaneous spreading (SS) process (**Figure** **1**a). Their conjugated structures enable comparable intermolecular interactions to the active layer materials with conjugated systems. To systematically evaluate their impact on the SS process, the structures (substituents, ring structures, or molecular symmetry) of aromatic small molecules are intentionally varied as differences.

Figure 1a shows the chemical structures of several solid additives that have been systematically studied, including molecules with monophenyl, diphenyl, and triphenyl rings. 1,4‐Diiodobenzene (DIB) features a benzene ring with two iodine atoms substituted at the para positions. The eutectic phase behavior induced by DIB enables the tailoring of the bulk and surface morphology of the active layer.^[^ ^21^ ^]^ The additive 1,4‐Dichlorobenzene (DCB), replacing the iodine atoms of DIB with smaller atomic radius chlorine atoms, possesses a fast removable property.^[^ ^22^ ^]^ Naphthalene (Nap), anthracene (An), and phenanthrene (PAT) are polycyclic aromatic compounds with extended conjugated systems. The high crystallinity of anthracene can help suppress excessive aggregation of fused‐ring nonfullerene acceptors during film formation.^[^ ^16^ ^]^ Similarly, due to the unique crystallinity, PAT shows a significant effect in inhibiting the over‐self‐aggregation of acceptors in the high‐boiling‐point solvent.^[^ ^15^ ^]^

It has been reported in the literature that such aromatic solid additives can exhibit strong intermolecular interactions with the donor/acceptor system, enabling morphology control and achieving ordered molecular stacking during the spin coating process.^[^ ^23^ ^]^ However, considering that the film‐forming kinetics are quite different between SS and the spin‐coating process, the role of these additives in the SS films is not yet established.

To understand the molecular interactions, we calculated the electrostatic potential surfaces (ESP) by using the DFT simulation at the b3lyp/def2svp level (Figure 1b). According to the computational results, the polyphenyl rings in Nap, An, and PAT showed positive values, indicating that these regions can form attractive interactions with electron donor sites. Literature shows that the nitrogen atom in the cyano group exhibits the minimum negative value in the Y6 molecule.^[^ ^21^ ^]^ Therefore, we speculate that the polyphenyl ring structure in aromatic additives can interact with the cyano group in Y6, thereby affecting the surface tension of the blend solutions and the shape of the SS‐films on the water surface.

The spontaneous spreading (SS) process of PM6:Y6 dissolved in o‐xylene with different solid additives is recorded with a camera (Figure S1, Supporting Information). The photographs of these liquid films after complete drying are summarized in Figure 1c. The droplets were observed to spread completely within 5 s, followed by gradual drying over 30 s. With DIB or DCB additives, many cracks gradually formed in the later drying time of the wet film, similar to the additive‐free control. While Nap and An additives prevented the appearance of cracks, the excessive solution accumulation at the edges hindered the formation of a uniform film. In contrast, the PAT additive enabled the formation of a complete and uniform film after complete drying, demonstrating the optimal shape on the water surface. The dried SS‐films on the water surface can be transferred to the ITO substrate (Figure 1d). The transfer methods are detailed in the experimental section. Among them, the SS‐PM6:Y6 films with Nap, An, and PAT were transferred as uniform thin films.

The surface tension characterization of solid additive solutions in o‐xylene revealed substantial differences in SS‐films (Figure S2, Supporting Information; Figure 1e). Notably, the PAT solution demonstrated the lowest surface tension of 26.5 mN m^−1^. This surface tension affects the spreading coefficient $\left(\vec{S}\right)$. Here, γ_1_ and γ_2_ represent the surface energies of water and PM6:Y6 solution, respectively. γ_12_ is the interfacial energy between the two solutions.^[^ ^4^ ^]^ The reduction in γ_2_ directly enhances the $\vec{S}$, as described by Equation (1). The relationship explains the improvement in SS‐film quality when PAT is introduced.

(1) $$\vec{S}=\gamma_{1}-\gamma_{2}-\gamma_{12}$$

The surface tension of solid additive films prepared by the spin‐coating method is subsequently studied (Figure 1f). The surface energy consists of a dispersion component (γd) and a polar component (γp), calculated using the contact angle of water and ethylene glycol (EG). The contact angles of these films are shown in Figure S3 (Supporting Information). As summarized in Table S2 (Supporting Information), the PAT film exhibited the lowest surface energy with a γp of 1.65 mN m^−1^ and γd of 24.72 mN m^−1^. In contrast, the other additive films showed reversed behavior, with γp ≈40 mN m^−1^ and γd ≈2 mN m^−1^. This result was consistent with the decrease in surface tension of o‐xylene solution after adding PAT (Figure 1e).

Given that dispersion forces are influenced by multiple factors, we cannot fully elaborate on all the reasons behind the strong dispersion force of PAT, and instead focus on the perspective of molecular symmetry.^[^ ^24^ ^]^ PAT belongs to the C_2V_ point group, with lower symmetry than DIB, DCB, An, and Nap, which belong to the D_2h_ point group. The dipole moments of each additive were obtained through simulation calculations, and the results show that only PAT has a weak dipole moment (0.0467 D), while the others are 0. The low symmetry of PAT leads to a slight unevenness in its electron cloud distribution, which in turn gives rise to this weak dipole moment. In addition to the dipole moment, low symmetry also increases the polarizability through the uneven distribution of the electron cloud, strengthens the interaction between the instantaneous dipole and the induced dipole of molecules, and ultimately, the synergistic effect manifests as an enhancement of the dispersion force.

**Figure 2:**
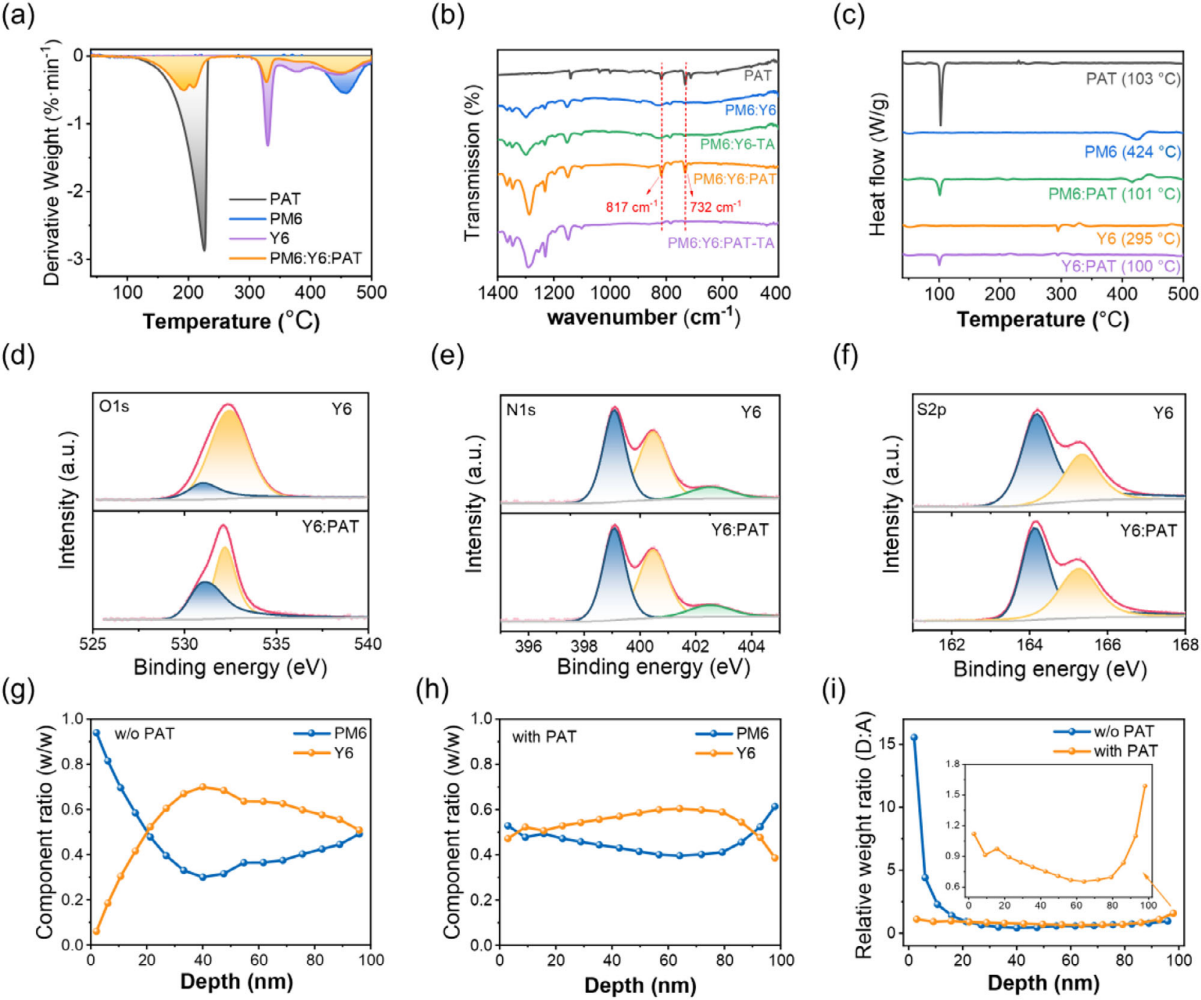
a) DTG curves of PAT, PM6, Y6, and PM6:Y6:PAT powder. b) FT‐IR spectra of the SS‐PM6:Y6 and SS‐PM6:Y6:PAT films without/with thermal annealing (100 °C for 2 min). c) DSC heating curves of PAT, PM6, PM6:PAT, Y6, and Y6:PAT powder at a heating rate of 10 °C min^−1^. XPS core level spectra of the d) O 1s, e) N 1s, and f) S 2p peaks for SS‐PM6:Y6 and SS‐PM6:Y6:PAT films. Vertical phase distribution curves of g) SS‐PM6:Y6 and h) SS‐PM6:Y6:PAT films. i) The relative weight ratio of D:A as a function of depth in SS‐films obtained from FLAS.

Due to the unique interfacial properties and excellent film‐forming ability, PAT was selected as a solid additive among the above solid additives, and the SS films prepared with the PAT additive were further studied. The thermogravimetric analysis (TGA) of PAT revealed a decomposition temperature (*T*_d_, 5% weight loss) of 156 °C (Figure S4, Supporting Information). The thermal stability of PM6, Y6, and the blend PM6:Y6 (1:1.2 w/w) was also systematically investigated through TGA (Figure S5, Supporting Information). The pure PM6 exhibited the highest thermal stability with a *T*_d_ of 426 °C, followed by Y6 (325 °C). The blend PM6:Y6 maintained a *T*_d_ of 325 °C, mirroring Y6's decomposition temperature. Notably, the incorporation of PAT at the same weight as Y6 significantly reduced the *T*_d_ of PM6, Y6, and PM6:Y6 to ≈160 °C. The derivative thermogravimetry (DTG) curves were obtained by differentiating the TGA curves (**Figure** **2**a). These curves revealed distinct endothermic peaks following a sequence as PAT (lowest stability) < Y6 < PM6 (highest stability). After adding PAT, the Δ*T*_d_ of Y6:PAT (179 °C) was smaller than that of PM6:PAT (267 °C), indicating a stronger intermolecular interaction between PAT and Y6, which may help PAT as a volatile additive to affect the crystallinity of Y6 during the annealing process.

Fourier transform infrared (FT‐IR) spectroscopy of the PM6:Y6:PAT further confirmed the volatilization of PAT after thermal annealing at 110 °C for 2 min, evidenced by the disappearance of PAT characteristic peaks at 732 and 817 cm^−1^ (Figure 2b). No new characteristic signals were detected after annealing under these conditions. Considering that PAT has a boiling point of 340 °C, the weight loss signal at *T*_d_ of 156 °C in the TGA is more likely attributed to the physical volatilization of phenanthrene rather than its chemical decomposition (Figure S4, Supporting Information).

These results demonstrate the suitability of PAT as a volatile additive under mild annealing conditions. In addition to volatility, the crystalline behavior of PAT was investigated by differential scanning calorimetry (DSC). As shown in Figure 2c, the DSC thermogram of pristine PAT exhibits a melting temperature (*T*_m_) of 103 °C with a melting enthalpy (Δ*H*_m_) of 42.9 J g^−1^. In contrast, Y6 and PM6 displayed significantly higher *T*_m_ values of 295 °C (Δ*H*_m_ = 3.1 J g^−1^) and 424 °C (Δ*H*_m_ = 29.0 J g^−1^), respectively. After blending with PAT, the characteristic peaks corresponding to Y6:PAT and PM6:PAT exhibited *T*_m_ of 100 and 101 °C, respectively. Combined with the absence of characteristic signals of new products in FT‐IR spectroscopy (Figure 2b), we speculate that the slight changes in peak intensity and position in the DSC heating curves result from the shift of PAT's characteristic signals rather than the formation of new melting peaks. Additionally, the Y6:PAT exhibited significantly lower Δ*H*_m_ (8.37 J g^−1^) compared to the PM6:PAT blend (17.2 J g^−1^). The greater Δ*H*_m_ suppression in Y6:PAT indicates that PAT more effectively suppresses the Y6's crystallization than PM6's.

The chemical interaction between PAT/PM6 and PAT/Y6 was examined through X‐ray Photoelectron Spectroscopy (XPS) analysis of O 1s, N 1s, and S 2p core levels. The results of peak position and area are summarized in Table S3 (Supporting Information). The O 1s XPS spectra of pristine Y6 exhibited two characteristic peaks at 531.0 eV (free C═O) and 532.3 eV (hydrogen‐bonded C═O) with an initial area ratio (*R*_531_/_532_) of 0.14, which increased significantly to 0.89 in the Y6:PAT blend, indicating PAT‐induced modulation of Y6's carbonyl groups (Figure 2d). In contrast, PM6 showed consistent O 1s peak ratios (*R*_531_/_532_ = 1.7) regardless of the addition of PAT (Figure S6a, Supporting Information).

Since PAT lacks oxygen functional groups, the observed O 1s signals at 530–532 eV in PAT film could potentially originate from surface‐adsorbed oxygen species during film processing (Figure S7, Supporting Information). This suggests that such surface oxygen might passivate hydrogen‐bonding sources in Y6, possibly contributing to the increased proportion of free C═O groups. In comparison, PM6's oxygen‐containing sites show no significant changes, which may be attributed to steric hindrance effects from the polymer backbone that could limit interactions with PAT. In contrast, N 1s in Y6, S 2p in PM6, and Y6 spectra exhibited negligible variations after adding PAT, which confirmed that PAT's interaction is mediated through oxygen coordination rather than nitrogen/sulfur participation (Figure 2e,f; Figure S6b, Supporting Information).

Film‐depth‐dependent Light Absorption Spectroscopy (FLAS) measurement was performed to investigate the vertical composition profiles of the active layer with PAT additive (Figure S8, Supporting Information). The SS‐films were transferred onto substrates with a PEDOT:PSS layer. The depth value of 0 corresponds to the water‐side of the film (close to PDINN after transfer), while the depth value of 100 represents the air‐side (close to PEDOT:PSS after transfer). The absorption spectra of individual sublayers were analyzed through linear combination fitting of neat component spectra. The vertical phase distribution curves of the SS‐PM6:Y6 and SS‐PM6:Y6:PAT films are shown in Figure 2g,h.

Figure 2i shows the relative weight ratio of donor:acceptor (D:A) as a function of depth in films. The PM6:Y6 film demonstrated a strong vertical segregation with the D:A ratio decreasing from 15 at the water‐side to 1 at the air‐side, indicating substantial accumulation of PM6 near the water surface. In contrast, the PM6:Y6:PAT film exhibited a relatively stable D:A ratio across the entire film depth, varying within the range of 0.5–1.5. Observing the enlarged view of this curve (inset in Figure 2i), PM6 was predominantly distributed near the PEDOT:PSS side, facilitating preferential contact between the hole transport layer and the donor for efficient hole transport.^[^ ^25^ ^]^ Thus, the addition of PAT mitigated the excessive accumulation of PM6 at the water interface and improved the vertical uniformity of the SS‐films.

**Figure 3:**
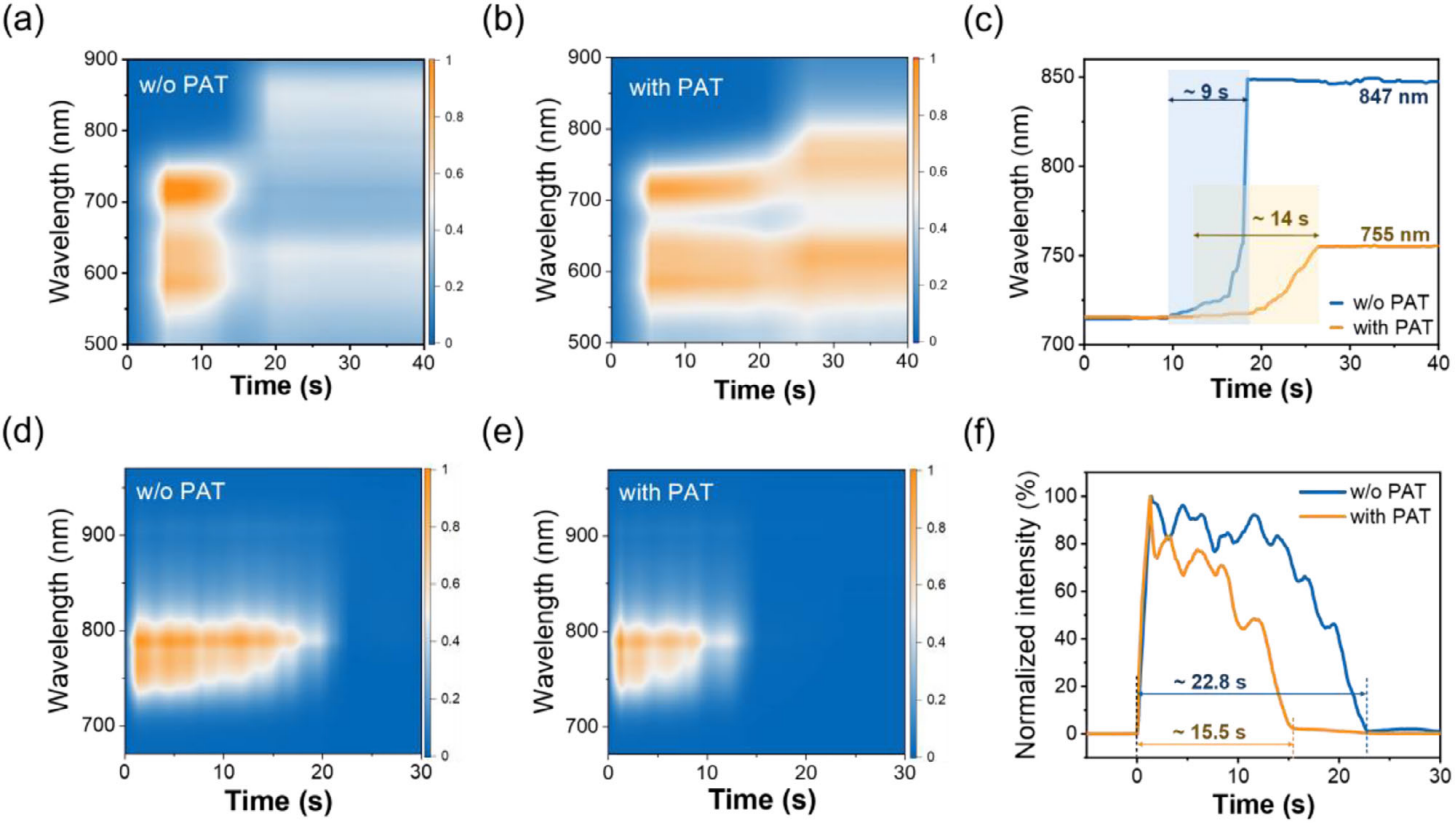
In situ time‐dependent a,b) absorption spectra, c) evolution of the main peak of Y6 absorption, d,e) fluorescence spectra, f) profiles of fluorescence at 790 nm for PM6:Y6 solution without and with PAT during SS process.

In situ absorption spectroscopy was subsequently employed to explore film‐forming kinetics. **Figure** **3**a,b presents the evolution of absorption spectra for the PM6:Y6 and PM6:Y6:PAT solutions during the SS process. The absorption intensity of the PM6:Y6 solution exhibited a rapid decline after the droplet spreading, which correlated with the observed crack formation on the water surface. However, the stability of the absorption intensity in PM6:Y6:PAT solution confirmed the role of PAT in stabilizing the SS‐film against cracking, which was consistent with the morphology improvement in Figure 1c. The changes in the position of Y6 peaks over time were extracted to reflect the speed of film formation (Figure 3c). The Y6 absorption peak shifted from 715 to 847 nm with a film‐forming time of ≈9 s for the PM6:Y6 solution. By contrast, the PM6:Y6:PAT solution demonstrated a lower peak shift from 715 to 755 nm and required a longer film‐forming time of ≈14 s. After adding PAT, the evolution of the absorption spectrum of PM6:Y6 solution was slower and more controllable. The lower peak shift indicates that PAT helps maintain molecular order and prevents excessive aggregation of the Y6. These results demonstrate that PAT significantly modifies the film‐forming kinetics during the SS processing.

The film‐forming kinetics were further investigated through fluorescence spectroscopy under 660 nm monochromatic light excitation, which specifically probes the solution‐phase emission ≈790 nm. Figure 3d,e presents the contour maps of fluorescence intensity evolution for PM6:Y6 and PM6:Y6:PAT solutions, where the minor spectral fluctuations originated from the water surface vibrations during the SS process. The emission profiles at 790 nm showed markedly different durations, with fluorescence persisting for 22.8 s in PM6:Y6 solution but only 15.5 s in PM6:Y6:PAT solution (Figure 3f). The reduced fluorescence lifetime in the PM6:Y6:PAT solution implied a more efficient exciton quenching, which may be due to the suppression of Y6 aggregation.

**Figure 4:**
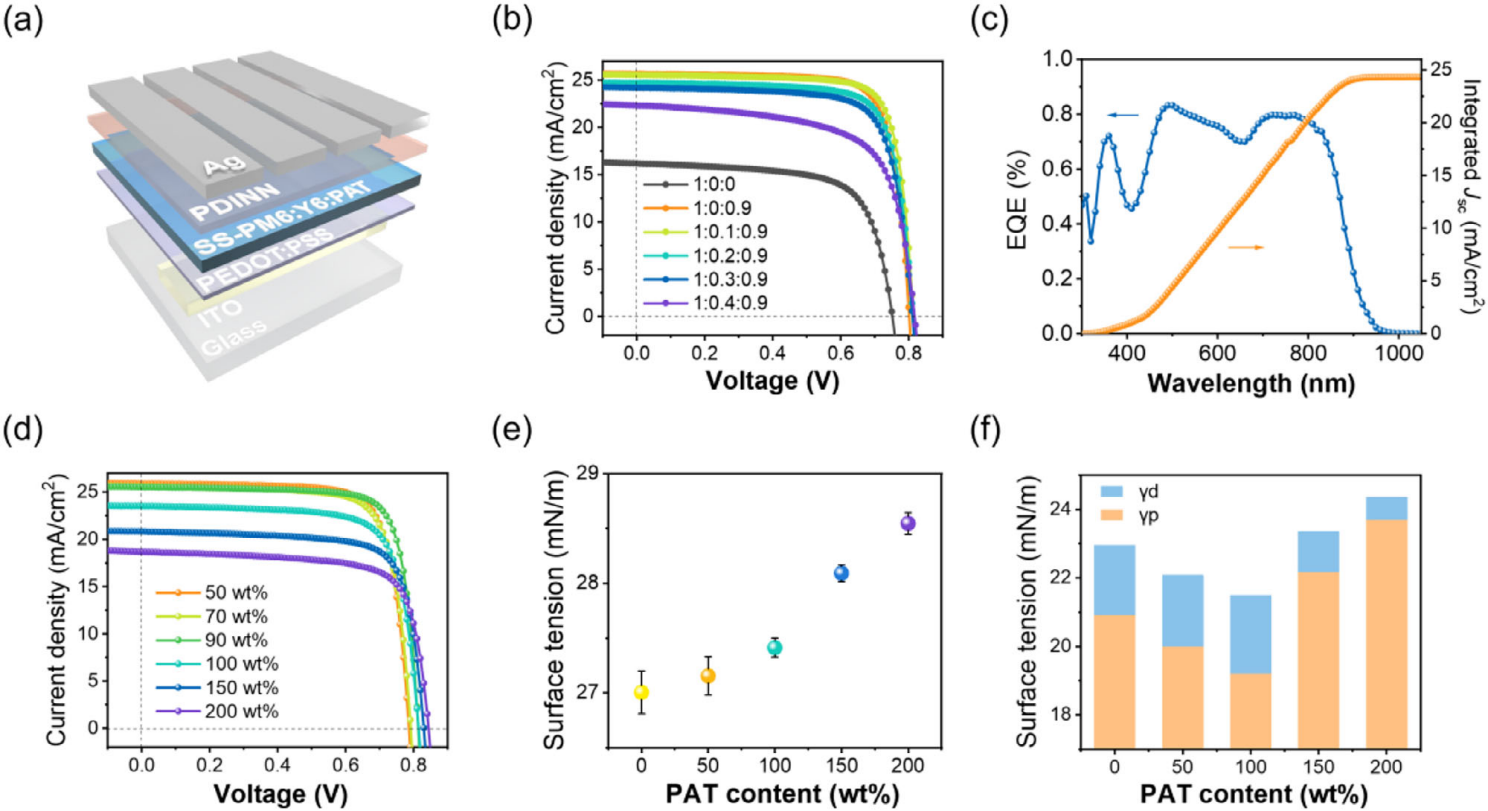
Device performance of SS‐OPVs. a) Schematic diagram of SS‐OPV structure. b) *J–V* curves of SS‐OPVs prepared by PM6:Y6:PC_71_BM with different PC_71_BM and PAT proportions. (The legend shows the mass ratio of Y6:PC_71_BM:PAT.) c) EQE spectrum and the corresponding integrated photocurrent of SS‐OPV with PM6:Y6:PC_71_BM:PAT film. d) *J–V* curves of SS‐OPVs, surface tension of e) the solutions, and f) the SS‐films on the water‐side for PM6:Y6:PC_71_BM:PAT with different PAT content.

The above systematic characterizations revealed that PAT effectively modulated the surface tension and film‐forming kinetics of PM6:Y6 dissolved in o‐xylene, thereby promoting the formation of uniform SS‐films on the water surface. The SS‐films were subsequently employed as active layers in OPV devices with the structure of ITO/PEDOT:PSS/SS‐PM6:Y6:PAT/PDINN/Ag, designated as SS‐OPVs in **Figure** **4**a. To achieve high‐performance devices, the solution concentration was increased to 22 mg mL^−1^ to obtain films with an appropriate thickness of ≈100 nm.

The SS‐OPV devices fabricated by PM6:Y6 without PAT exhibited a poor power conversion efficiency (PCE) of ≈8% (Figure 4b; **Table** **1**). This SS‐PM6:Y6 film showed nonideal film thickness and morphology (Figure S9a, Supporting Information). After adding 90 wt.% PAT, the large‐area uniform film of SS‐PM6:Y6:PAT enabled the devices with a dramatically enhanced PCE of 16.1%. The open‐circuit voltage (*V*_OC)_, short‐circuit current density (*J*_SC_), and fill factor (FF) were all improved (Figure S9b, Supporting Information). On this basis, we compared the SS process of PM6:Y6 solutions with different proportions of PC_71_BM. The *V*_OC_ of the SS‐OPV device was improved from 0.802 to 0.814 V by introducing 10 wt.% PC_71_BM, and the device PCE was increased to 16.4%. The external quantum efficiency (EQE) spectrum indicated that the integrated *J*_SC_ of 24.3 mA cm^−2^ matched well with the measured *J*_SC_ of 25.5 mA cm^−2^ (Figure 4c).

**Table 1:** The photovoltaic parameters of SS‐OPVs made by SS‐PM6:Y6:PC_71_BM:PAT films with different PC_71_BM and PAT proportions.

Y6:PC_71_BM:PAT	*V*_OC_ [V]	*J*_SC_ [mA cm^2^]	FF [%]	PCE [%]
1:0:0	0.741 ± 0.006 0.752	15.8 ± 0.36 16.2	67.3 ± 1.50 68.8	7.88 ± 0.48 8.36
1:0:0.9	0.801 ± 0.001 0.802	25.7 ± 0.16 25.6	77.1 ± 0.75 78.2	15.8 ± 0.15 16.1
1:0.1:0.9	0.811 ± 0.004 0.814	25.4 ± 0.24 25.5	78.1 ± 0.76 78.9	16.1 ± 0.38 16.4
1:0.2:0.9	0.811 ± 0.002 0.813	24.6 ± 0.34 24.7	76.4 ± 0.76 76.7	15.2 ± 0.14 15.4
1:0.3:0.9	0.812 ± 0.003 0.811	24.3 ± 0.26 24.2	75.0 ± 0.12 75.1	14.8 ± 0.20 14.7
1:0.4:0.9	0.816 ± 0.006 0.817	22.4 ± 0.13 22.3	66.6 ± 0.19 66.7	12.2 ± 0.17 12.1

*Statistical values obtained from ten independent devices.

The effects of PAT proportion on device performance were also studied. The *J–V* results shown in Figure 4d and Table S4 (Supporting Information) exhibited a positive correlation between PAT proportion and *V*_OC_. The investigation of surface tension demonstrated that increasing PAT proportion in PM6:Y6 solution led to progressively higher surface tension values (Figure 4e; Figure S10, Supporting Information). However, the overall variation remained relatively small for PAT less than 100 wt.% (27.1–27.3 mJ m^−1^). The photographs in Figure S11 (Supporting Information) revealed that PAT proportions between 50 wt.% and 90 wt.% enabled the formation of sufficiently uniform SS‐films. The water‐side surface tension of these SS‐films exhibited a distinct non‐monotonic dependence on PAT proportion, reaching a minimum value of 21.5 mJ m^−1^ at 100 wt.% PAT (Figure 4f; Figure S12 and Table S5, Supporting Information).

**Figure 5:**
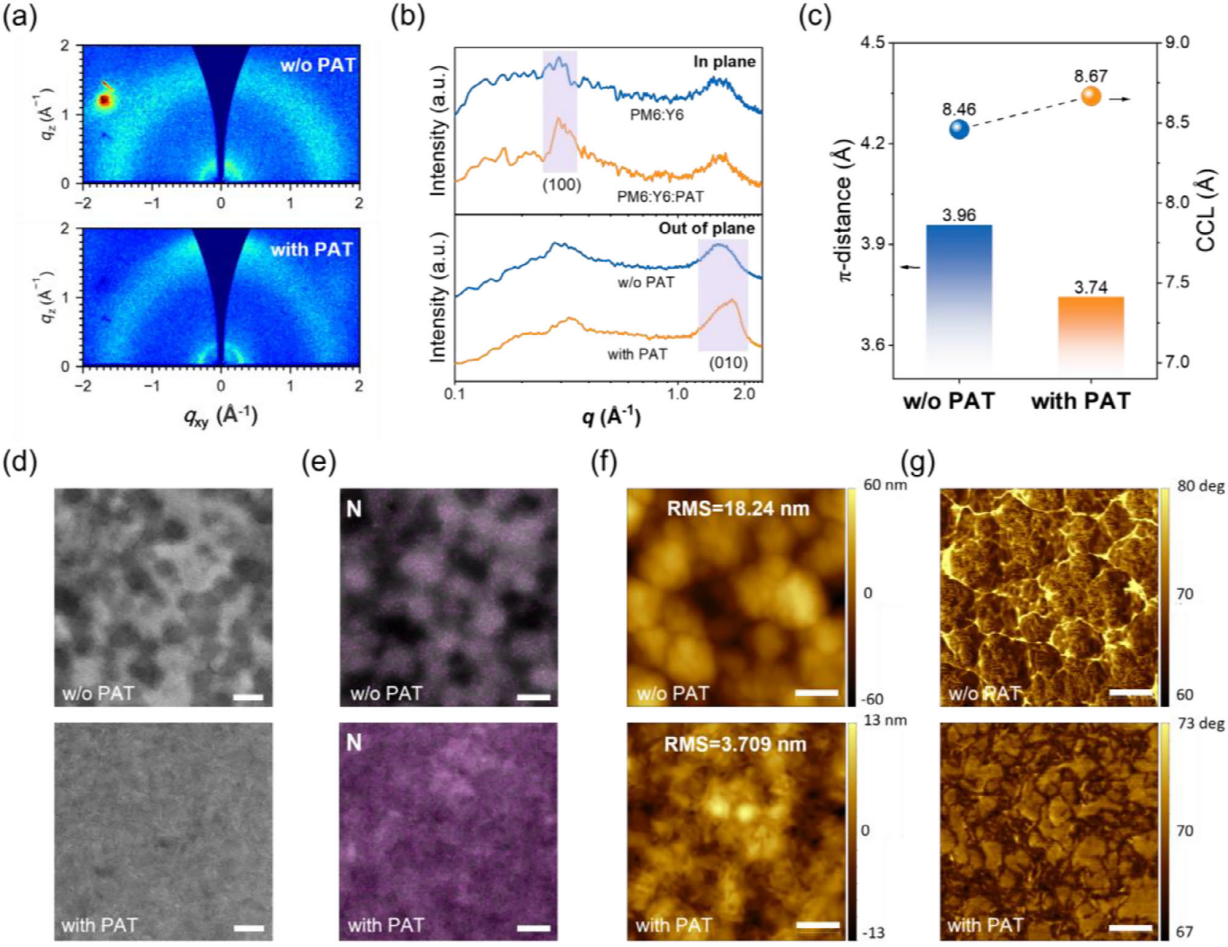
Characterization of SS‐film morphology. a) 2D GIWAXS patterns, b) corresponding 1D GIWAXS line‐cut profiles of the in‐plane and out‐of‐plane directions, c) π–π stacking distance and crystal coherence length (CCL), d) SEM image, e) nitrogen distribution diagram, f) AFM height images, g) AFM phase images of the SS‐PM6:Y6:PC_71_BM without and with PAT films. (The scale bar is 200 nm.).

The molecular packing characteristics of SS‐films were investigated by grazing‐incidence wide‐angle X‐ray scattering (GIWAXS). **Figure** **5**a presents the 2D GIWAXS patterns, while Figure 5b displays the corresponding 1D line‐cut profiles along both in‐plane and out‐of‐plane directions.

The SS‐film with PAT exhibited a slightly stronger diffraction peak at the (100) in the in‐plane direction compared to the film without PAT, suggesting that PAT promotes the side‐chain packing of molecules. The (010) peaks, corresponding π–π stacking distance (d010), full width at half maximum (FWHM), and crystal coherence length (CCL) of these SS‐films are summarized in Table S6 (Supporting Information). In both films with and without PAT, the (010) peak appeared in both the out‐of‐plane and in‐plane directions, indicating the coexistence of face‐on orientation (π–π stacking is perpendicular to the substrate) and edge‐on orientation (π–π stacking is parallel to the substrate). This implies that in the SS process, some molecules adopt an edge‐on orientation to optimize in‐plane charge transport, while others retain a face‐on orientation to enhance vertical charge collection.

Notably, in the out‐of‐plane direction, the (010) peak position shifted from 1.59 to 1.68 Å^−1^ with marginally increased intensity after adding PAT to the SS‐film. This demonstrates that PAT facilitates tighter packing (reduced interlayer spacing) and slightly improved ordering of the face‐on π–π stacking. Similar phenomena affecting molecular stacking were also mentioned in articles studying the introduction of a third component to improve the PCE in bulk heterojunction or p‐i‐n structure OPVs.^[^ ^26^, ^27^, ^28^ ^]^ In combination with the phenomenon of decreased Δ*H*_m_ after adding PAT in Figure 2c, it can be observed that even though the long‐range order of Y6 is disrupted, it still form more regular and tighter short‐range arrangements locally (small‐sized but highly ordered crystalline domains), increasing the coherence length.

To understand the influence of the PAT on the phase separation morphology, we performed scanning electron microscopy (SEM) and atomic force microscopy (AFM) analysis on SS‐films without and with PAT. The SEM imaging in Figure 5d combined with elemental mapping of nitrogen in Figure 5e provided the phase separation behavior of the active layer. Since nitrogen (N) is exclusively present in the Y6, the N‐element mapping clearly distinguished the donor and acceptor domains, with dark regions representing PM6 and bright regions representing Y6. The SS‐PM6:Y6:PC_71_BM film exhibited severe phase segregation with distinct Y6‐rich domains of ≈100 nm. The SS‐PM6:Y6:PC_71_BM film displayed substantial surface roughness with a root‐mean‐square (RMS) value of 18.24 nm (Figure 5f), accompanied by excessively large aggregate formation in Figure 5g.

In contrast, the SS‐film with PAT demonstrated homogeneous distribution of these components without observable aggregation. The RMS value of the SS‐PM6:Y6:PC_71_BM:PAT film was significantly reduced to 3.709 nm without excessively large clusters. The phase images further revealed that the PAT promoted tighter molecular packing and more uniform phase distribution in the active layer. This morphological improvement directly correlates with enhanced device performance, as the improved phase morphology facilitates efficient exciton dissociation and charge transport while minimizing recombination losses.

**Figure 6:**
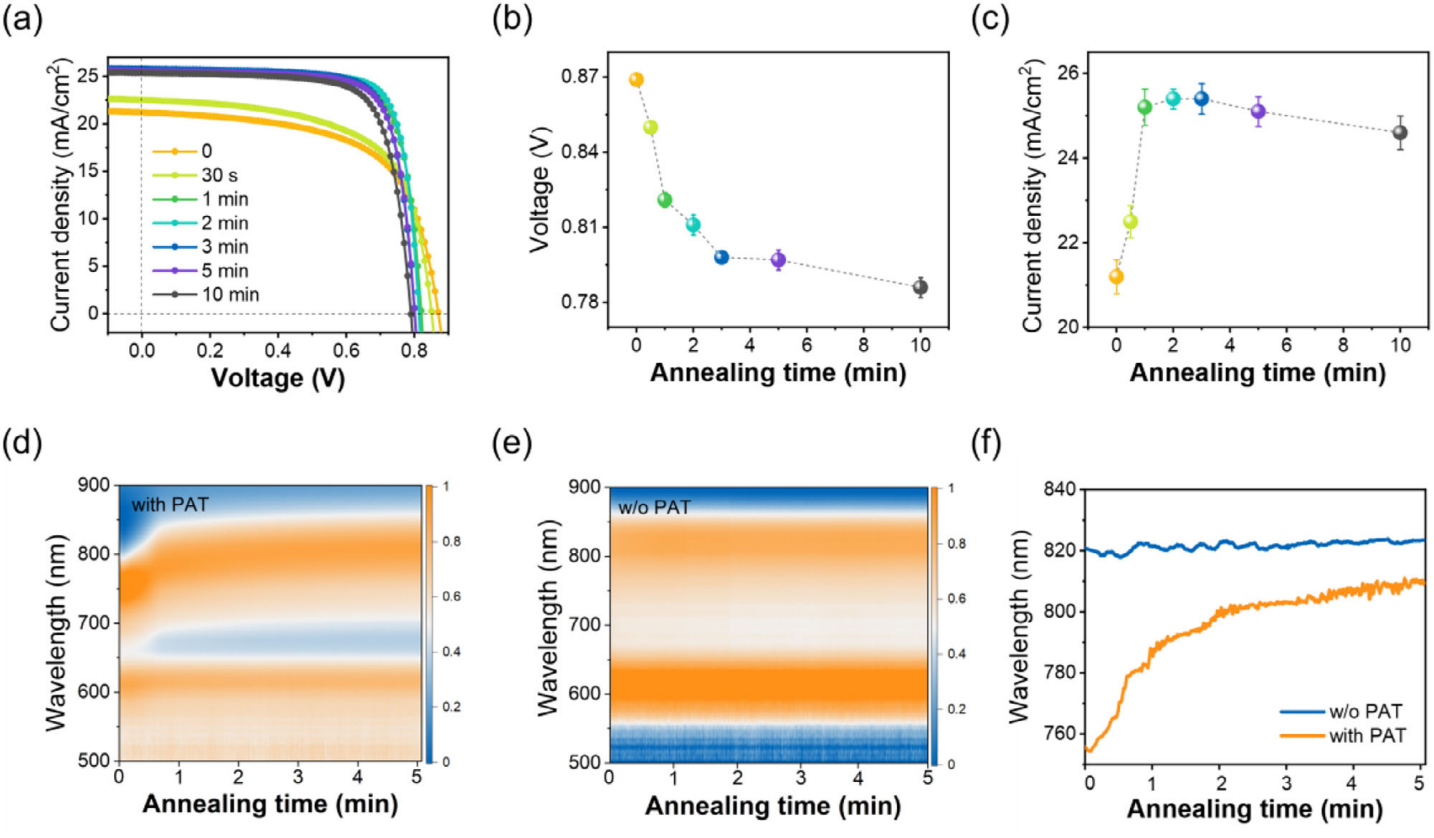
a) *J–V* curves of SS‐OPVs with PAT after different annealing times at 110 °C. Variation of b) *V*_OC_ and c) *J*_SC_ with annealing time. In situ time‐dependent absorption spectra of d) SS‐PM6:Y6:PC_71_BM:PAT and e) SS‐PM6:Y6:PC_71_BM films during annealing process. f) Evolution of the main peak of Y6 absorption during the annealing process.

Considering the volatility of the PAT, we further studied the effect of thermal annealing conditions on the device performance. As shown in Figure S13 and Table S7 (Supporting Information), the higher the annealing temperature of the SS‐PM6:Y6:PC_71_BM:PAT film, the lower the *V*_OC_. The *J*_SC_ reached a maximum value of 25.5 mA cm^−2^ when annealed at 110 °C, and the PCE of the SS‐OPV prepared under these conditions reached 16.4%. The photovoltaic characteristics of the devices with different annealing times at 110 °C are shown in **Figure** **6**a and Table S8 (Supporting Information). The complementary study showed that the effect of annealing temperature on device PCE was consistent with annealing time. The *V*_OC_ exhibited a continuous decrease with prolonged annealing time (Figure 6b), while the *J*_SC_ showed a nonmonotonic behavior, reaching optimal values within the 1–5 min range before declining (Figure 6c). These investigations established 110 °C for 2 min as the recommended annealing condition for SS‐PM6:Y6:PC_71_BM:PAT film, balancing *V*_OC_ and *J*_SC_ to achieve the best performance.

To elucidate the mechanism of annealing‐dependent device performance, we conducted the in situ time‐dependent absorption test for SS‐film during annealing. Figure 6d,e presents the spectral evolution of SS‐PM6:Y6:PC_71_BM films with and without PAT during annealing 5 min at 110 °C. While the PM6 absorption remained stable in both films, the Y6 absorption exhibited markedly different behavior after adding PAT. The peak position remained constant at 820 nm in the SS‐film without PAT, but progressively red‐shifted from 755 to 810 nm in the SS‐film with PAT during annealing (Figure 6f). The gradual red shift indicated progressive molecular ordering and enhanced π–π interactions of Y6, which was consistent with the *J*_SC_ improvement. In contrast, the static absorption of the SS‐film without PAT suggests that the pre‐existing aggregation could not be further optimized by thermal annealing, explaining its inferior performance. These results demonstrate that PAT alters the thermal reorganization dynamics of Y6, leading to the optimal device performance under the recommended condition of 110 °C for 2 min.

## 3 Conclusion

This study demonstrates that the aromatic solid additives can influence the film uniformity and molecular stacking of the active layer in the SS process by regulating surface tension and film‐forming kinetics. By comparing aromatic molecules with different substituents, conjugated structures, or symmetries, it is found that PAT, a triphenyl ring molecule with weak symmetry and no substituents, exhibits lower surface tension. The PAT extended the solution‐to‐solid transition time while accelerating exciton quenching. The PAT‐additive SS‐films with uniform phase distribution and excellent crystallinity were successfully applied in OPV devices. Notably, a remarkable PCE of 16.4% along with *V*_OC_ of 0.814 V, *J*_SC_ of 25.5 mA cm^−2^, and FF of 78.9% was recorded in the o‐xylene‐processed OPV device, which was much higher than the nonadditive control device with PCE of 8.8%. The thermal volatility of PAT enabled its removal during mild annealing (110 °C for 2 min), which affected the molecular stacking of Y6 and modulated the device performance. As the first systematic investigation of solid additives' impact on the SS process, the findings of PAT open a new possibility for developing high‐performance SS‐OPV devices through additive engineering.

The solid additives provide a complementary pathway to conventional liquid additives during the SS process. With their structurally designable backbones and moderate presence time, solid additives can introduce an additional dimension of regulation for film‐forming kinetics and phase separation behavior. With the same functions and properties in the spin‐coating process, the ideal solid additives generally feature planar backbones that promote ordered molecular packing. They have moderate volatility to regulate the film formation process while ultimately evaporating without leaving a residue. Good solubility and chemical inertness are desirable to ensure homogeneous distribution and minimize side reactions. Compared with the spin‐coating process, the influence of the surface energy of the active layer precursor solution caused by solid additives needs to be additionally evaluated in the SS process. In addition, tailored substituents (halogen, heteroatom, or alkyl groups) can further influence film crystallization behavior. These features provide essential regulation during the solution‐to‐solid transition of active layers, leading to controlled phase separation, improved molecular packing, and superior voltaic performance.

## 4 Experimental Section

### Materials

Patterned ITO glass substrates were purchased from Liaoning Youxuan Tech. Clevios P VP Al 4083 was purchased from Heraeus. Polymer donors PM6 (cas: 1802013‐83‐7), non‐fullerene acceptor Y6 (cas: 2304444‐49‐1), fullerene acceptor PC_71_BM (cas: 609771‐63‐3), and electron transport layer PDINN (cas: 1020180‐01‐1) were purchased from Solarmer. 1,4‐Diiodobenzene (DIB) was purchased from Shanghai yuanye Bio‐Technology Co., Ltd. 1,4‐Dichlorobenzene (DCB) was purchased from J&K Scientific. Anthracene (An) was purchased from Beijing InnoChem Science Technology Co., Ltd. Naphthalene (Nap) was purchased from Meryer (Shanghai) Biochemical Technology Co., Ltd. Phenanthrene (PAT) was purchased from Bide Pharm.

### Spreading and Transfer Process

A blend solution was prepared by dissolving PM6, Y6, and solid additive in o‐xylene at a weight ratio of 1:1.2:1.2, with PM6 concentration of 5 mg mL^−1^. The mixture was stirred at 80 °C for 1 h to ensure complete dissolution. A 25 µL aliquot of the solution was vertically dispensed onto the center of the water surface (23 °C) in a 65 mm diameter Petri dish. The SS‐film rapidly spread and dried slowly under ambient conditions (25 °C, ≈50% relative humidity). After complete drying, the substrate was sucked via vacuum suction pen and stamped obliquely on the film to avoid bubbles. After the edge of SS‐film was cut, the substrate was lifted obliquely. The entire process was conducted in a well‐ventilated open‐air condition.

### Fabrication of SS‐OPVs

SS‐OPVs with the structure of glass/ITO/PEDOT:PSS/SS‐PM6:Y6/PDINN/Ag were prepared with the following fabrication process. PEDOT:PSS diluted (1:1 vol%) with deionized water was spin‐coated on the plasma‐treated ITO glass (10.5 W for 2 min) at 4 krpm for 30 s, annealing at 120 °C for 5 min. The PM6:Y6 weight ratio was kept at 1:1.2 with PM6 concentration of 10 mg mL^−1^ in o‐xylene. The optimized weight ratio of PC_71_BM to Y6 was 10 wt.%. The optimized weight ratio of PAT to Y6 was 90 wt%. The optimized volume ratio of 1‐Chloronaphthalene was 0.5 vol%. The mixture was stirred at 80 °C for 1 h to ensure complete dissolution. In SS process, a 25 µL blend solution was dropped onto the water surface in a 65 mm diameter petri dish. After the SS‐film dried, the film was stamped onto the PEDOT:PSS‐coated layer. The optimal annealing condition was 110 °C for 2 min in air. After that, PDINN (1 mg mL^−1^ in methanol) was spin‐coated on the SS‐film at 3 krpm for 20 s in a glove box. Finally, a 60 nm Ag layer was deposited by thermal evaporation at a speed of 0.5 Å s^−1^. The device area of 9.2 mm^2^ was defined by the cross‐area of ITO and Ag electrodes.

### Characterization of Films and Devices

Contact angles of film and surface energy of solution were tested by OCA15EC. TGA and DSC were recorded on a TGA/DSC Simultaneous Thermal Analyzer (Mettler‐Toledo, TGA/DSC 3+/1600 HT). FT‐IR measurement of the SS‐film was conducted on a Bruker Vertex 80 V & Hyperion 3000 spectrometer. XPS was obtained by microfocus X‐ray photoelectron spectroscopy (Thermo Scientific, Nexsa G2). FLAS was performed by a PDU‐400 spectrometer from Shaanxi Puguangweishi Technology Co., Ltd. Time‐dependent in situ UV–Vis–NIR spectra were conducted on a DU‐300 dynamic spectrometer in transmission mode (Xenon lamp with wavelength from 190 to 1100 nm) and fluorescence mode (monochromatic light at 660 nm) with a time interval of 0.05 s from Shaanxi Puguangweishi Technology Co., Ltd. The *J–V* curve was obtained with a Keithley 2400 source, under the simulated AM 1.5 G illumination by 450 W Xenon‐lamp (Newport, M94043A). The device test area was 3 mm^2^ defined by a metal aperture mask. EQE spectra were achieved by a Newport EQE system (Quantx‐300) without any filter. GIWAXS was tested by Bruker D8 Venture & Bruker Nanostar. TEM images and elemental mapping were performed by Thermo Fisher Scientific Talos L120C G2. AFM image was obtained by Oxford Instruments from the Jupiter XR system.

## Conflict of Interest

The authors declare no conflict of interest.

## Author Contributions

H.D. carried out the experiments and collected the data. H.D. and D.L. conceived the experiments and prepared the manuscript. All authors contribute to the discussion of the results and revision of the manuscript. D.L. directed the study.

## Supporting information



Supporting Information

## Data Availability

The data that support the findings of this study are available in the supplementary material of this article.
