# Supplementary material for: Solid Additives for Spontaneously Spreading‐Processed Organic Photovoltaics
Source: Adv Sci (Weinh). 2025 Aug 26;12(43):e12384. doi: 10.1002/advs.202512384 (PMC12631901; doi:10.1002/advs.202512384)
Supplement: Supplementary file 1 — Supporting Information [file ADVS-12-e12384-s001.docx]

**Supporting Information**

**Solid Additives for Spontaneously Spreading-Processed Organic Photovoltaics**

*Huitong Deng^1,2^, Qianqing Jiang^2*^, Dianyi Liu^1,2,3,4,5*^*

^1^ Zhejiang University, Hangzhou, Zhejiang 310027, China.

^2^ Zhejiang Key Laboratory of 3D Micro/Nano Fabrication and Characterization, Research Center for Industries of the Future, Department of Electronic and Information Engineering, School of Engineering, Westlake University, Hangzhou, Zhejiang 310030, China.

^3^ Westlake Institute for Optoelectronics, Hangzhou, Zhejiang 311421, China.

^4^ Division of Solar Energy Conversion and Catalysis at Westlake University, Zhejiang Baima Lake Laboratory Co., Ltd., Hangzhou, Zhejiang 310000, China.

^5^ Westlake Optoelectronic Technology Co., Ltd., Hangzhou, Zhejiang 310024, China.

Corresponding Author

Dr. Qianqing Jiang

*Email: jiangqianqing@westlake.edu.cn

Dr. Dianyi Liu

* Email: liudianyi@westlake.edu.cn


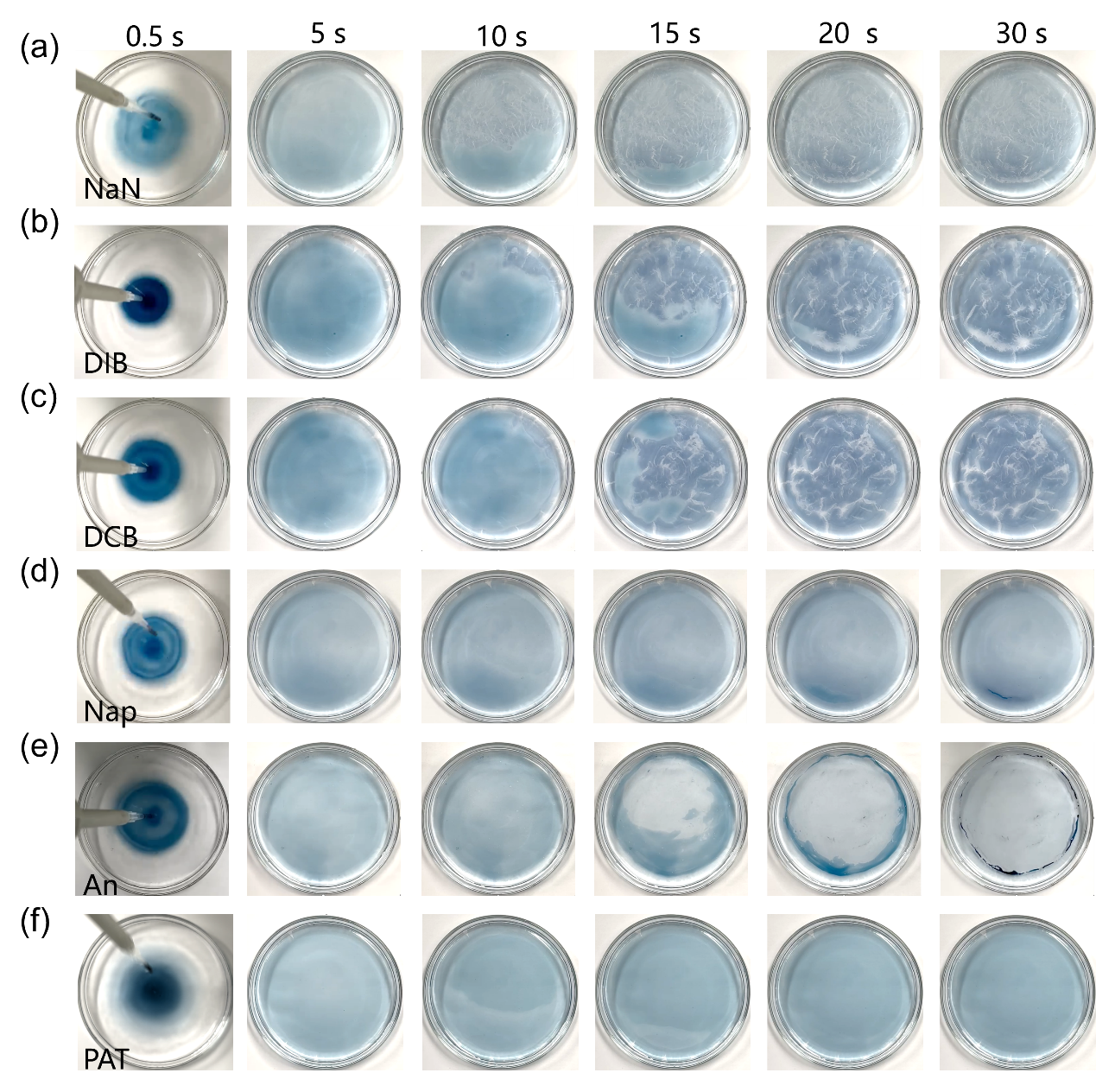


# **Figure S1.** Spreading process of PM6:Y6 with different solid additives on the water surface at different time.


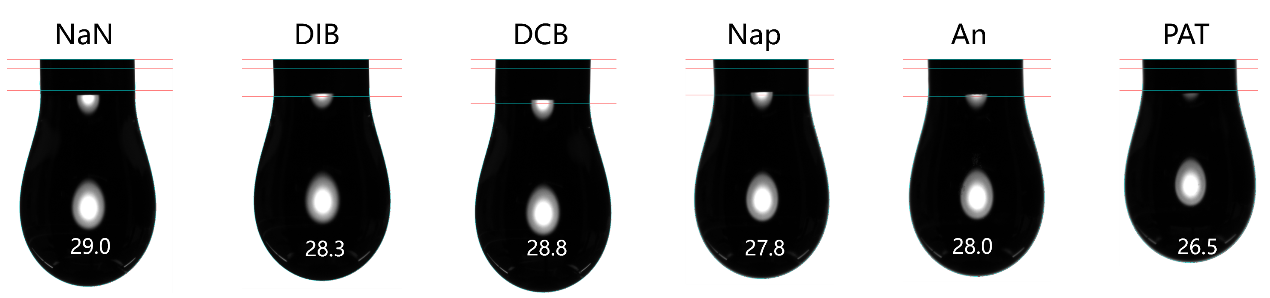


# **Figure S2.** Surface tension measurements of the solid additive dissolved in o-xylene at a concentration of 12 mg/mL. (The number is the measured value ​​of surface tension, the unit is mN/m.)


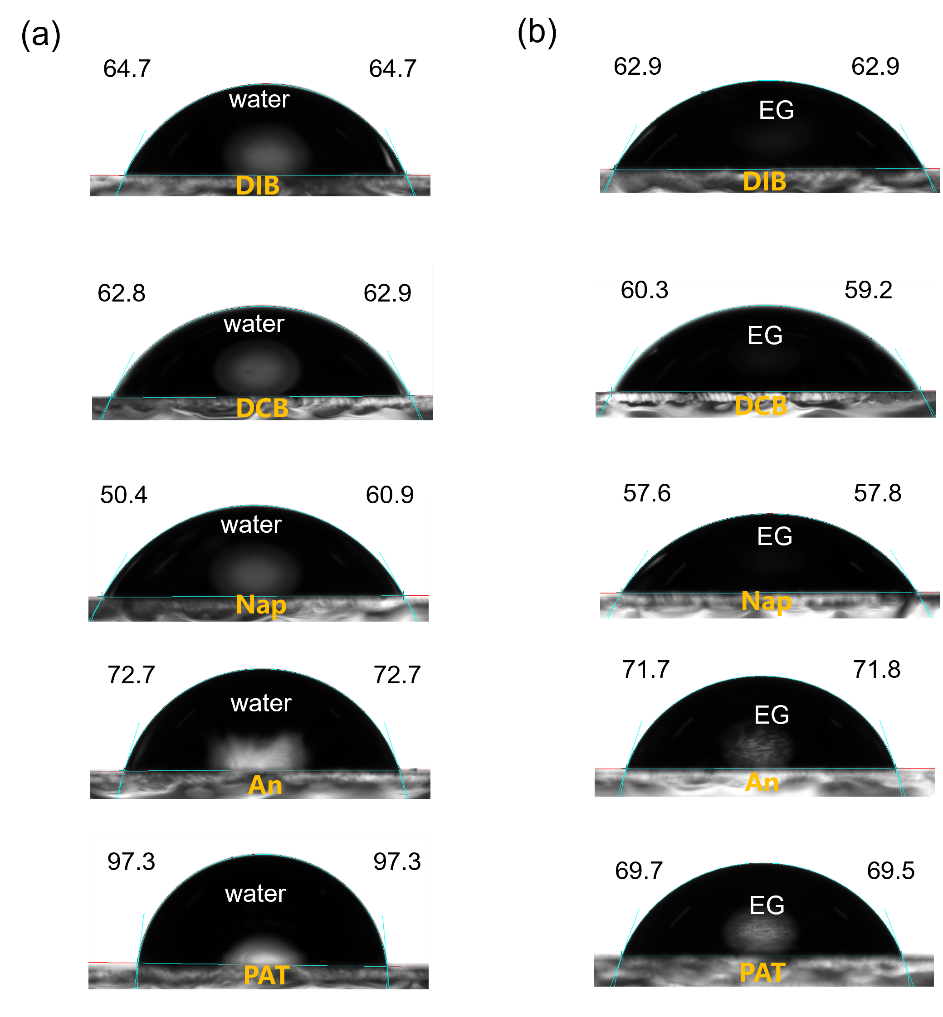


# **Figure S3.** Contact angles of (a) water, and (b) EG on different solid additive films.


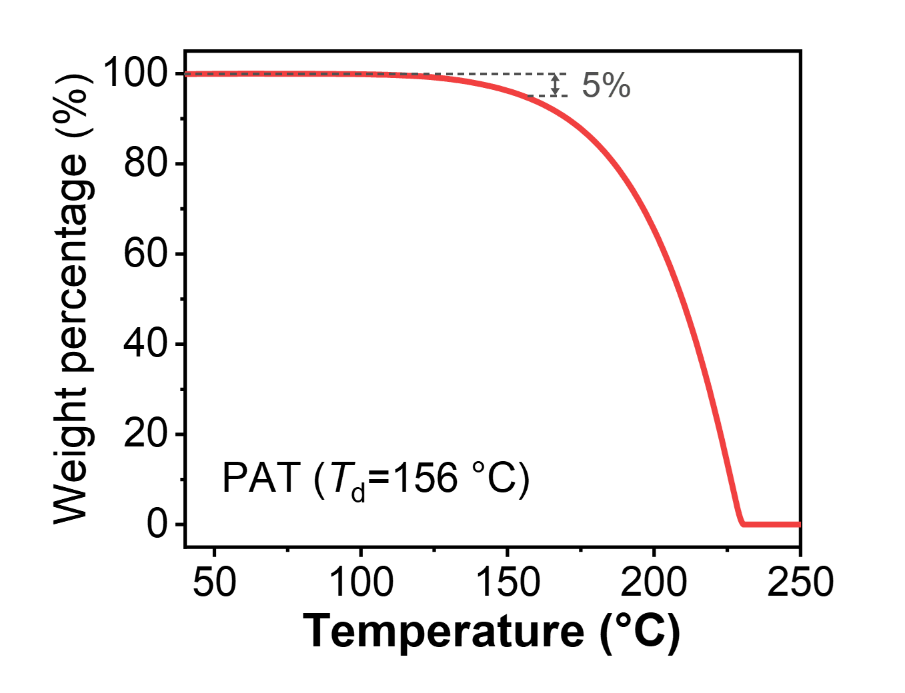


# **Figure S4.** TGA plot of PAT at a scan rate of 10 °C/min under nitrogen atmosphere.

#
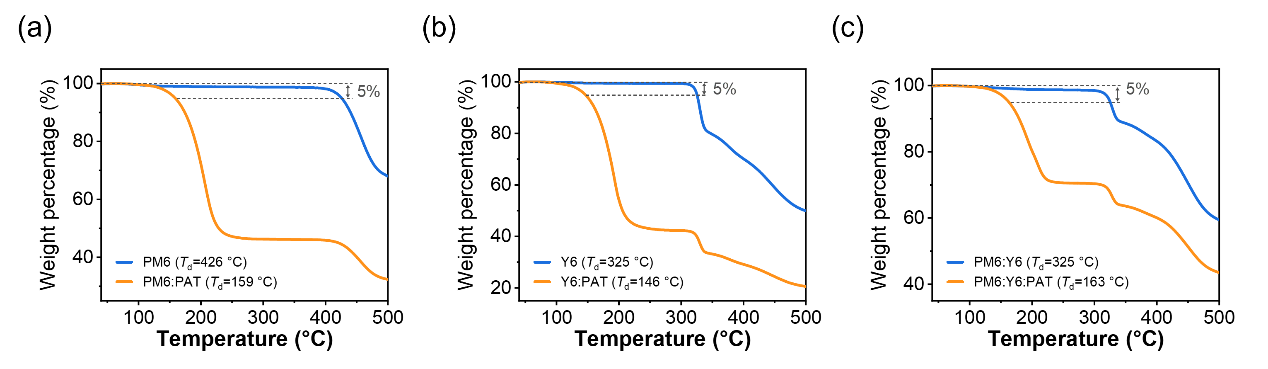
**Figure S5.** TGA plot of (a) PM6, (b) Y6, and (c) PM6:Y6 (1:1.2, w/w) without/with PAT at a scan rate of 10 °C/min under nitrogen atmosphere. (Y6:PAT=1:1, w/w)

#
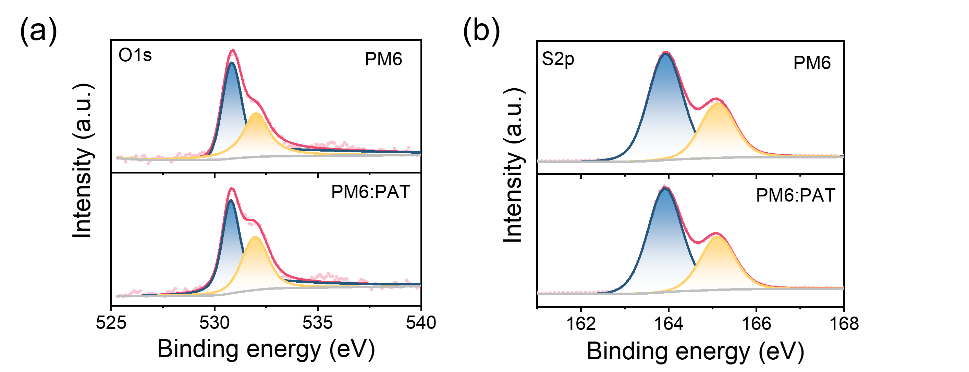
**Figure S6.** XPS core level spectra of the (a) O 1s and (b) S 2p peaks for SS- PM6 and SS-PM6:PAT films.

#
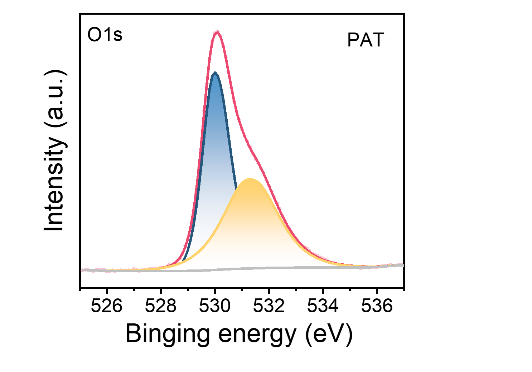
**Figure S7.** XPS core level spectra of the O 1s for PAT film.


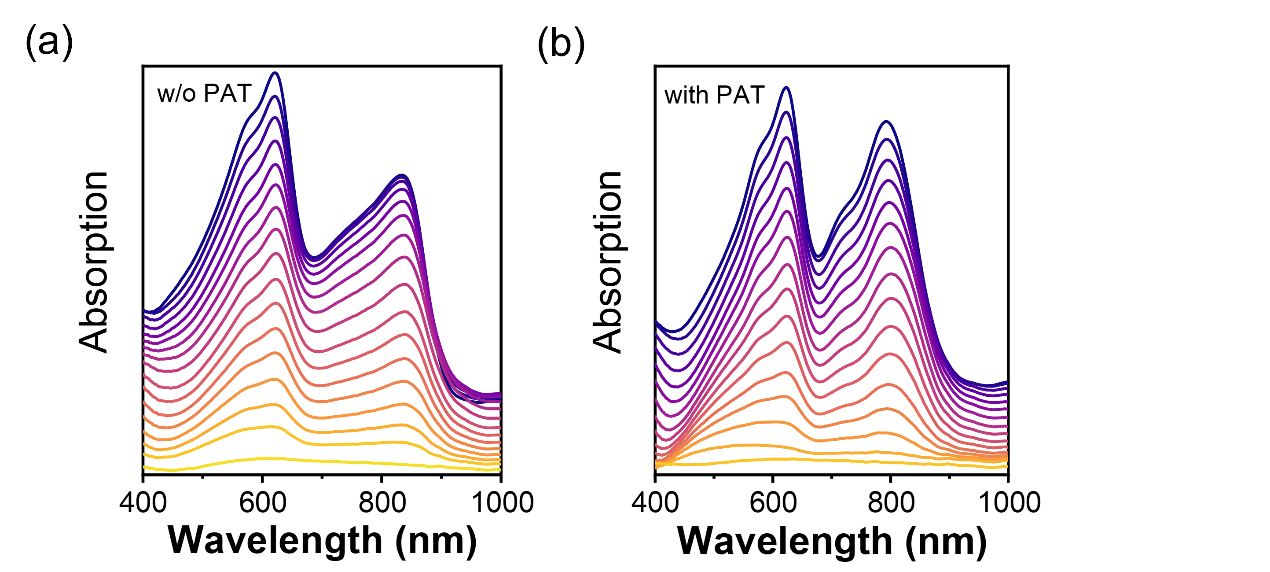


# **Figure S8.** Film-depth-dependent light absorption spectra (FLAS) of PM6:Y6 and PM6:Y6:PAT films.


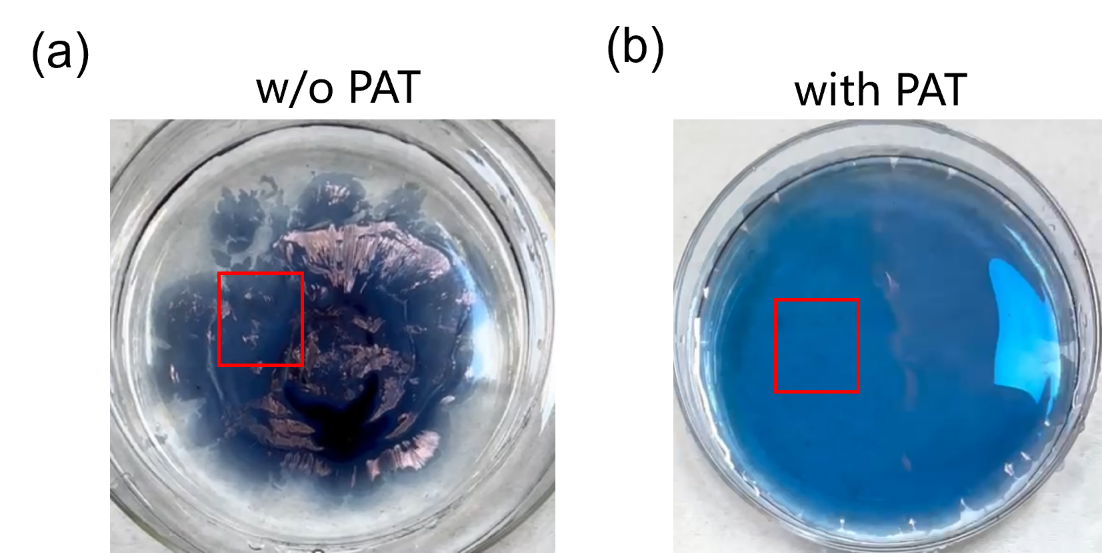


# **Figure S9.** Photographs of PM6:Y6 and PM6:Y6:PAT solutions with concentration of 22 mg/mL after spreading on the water surface. (The area within the red frame is the SS-film transferred onto the device.)

# **
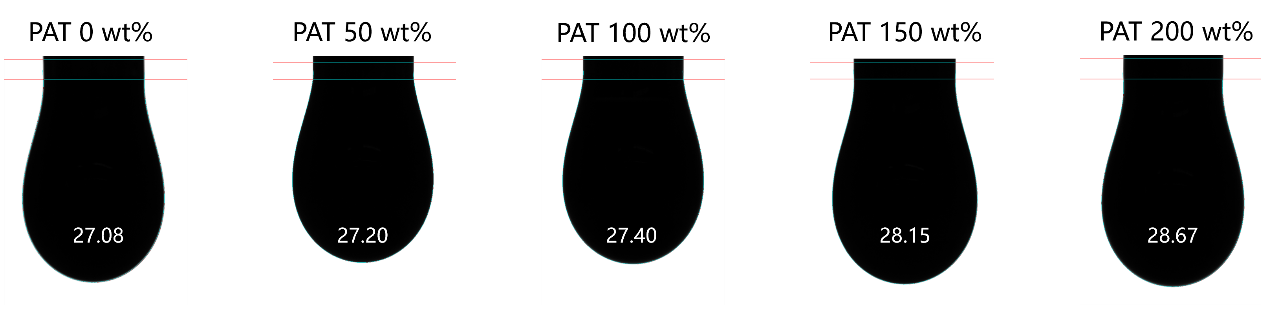
Figure S10.** Surface tension measurements of PM6:Y6:PC_71_BM solution with different PAT proportion. (The number is the measured value ​​of surface tension, the unit is mN/m.)

# **
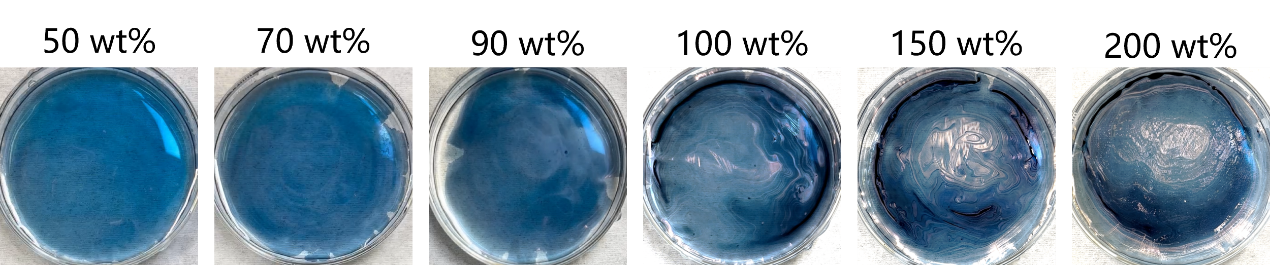
Figure S11.** Photographs of SS-PM6:Y6:PC_71_BM:PAT films with different PAT proportion after spreading on the water surface. (The diameter of petri dish is 65 mm.)

# **
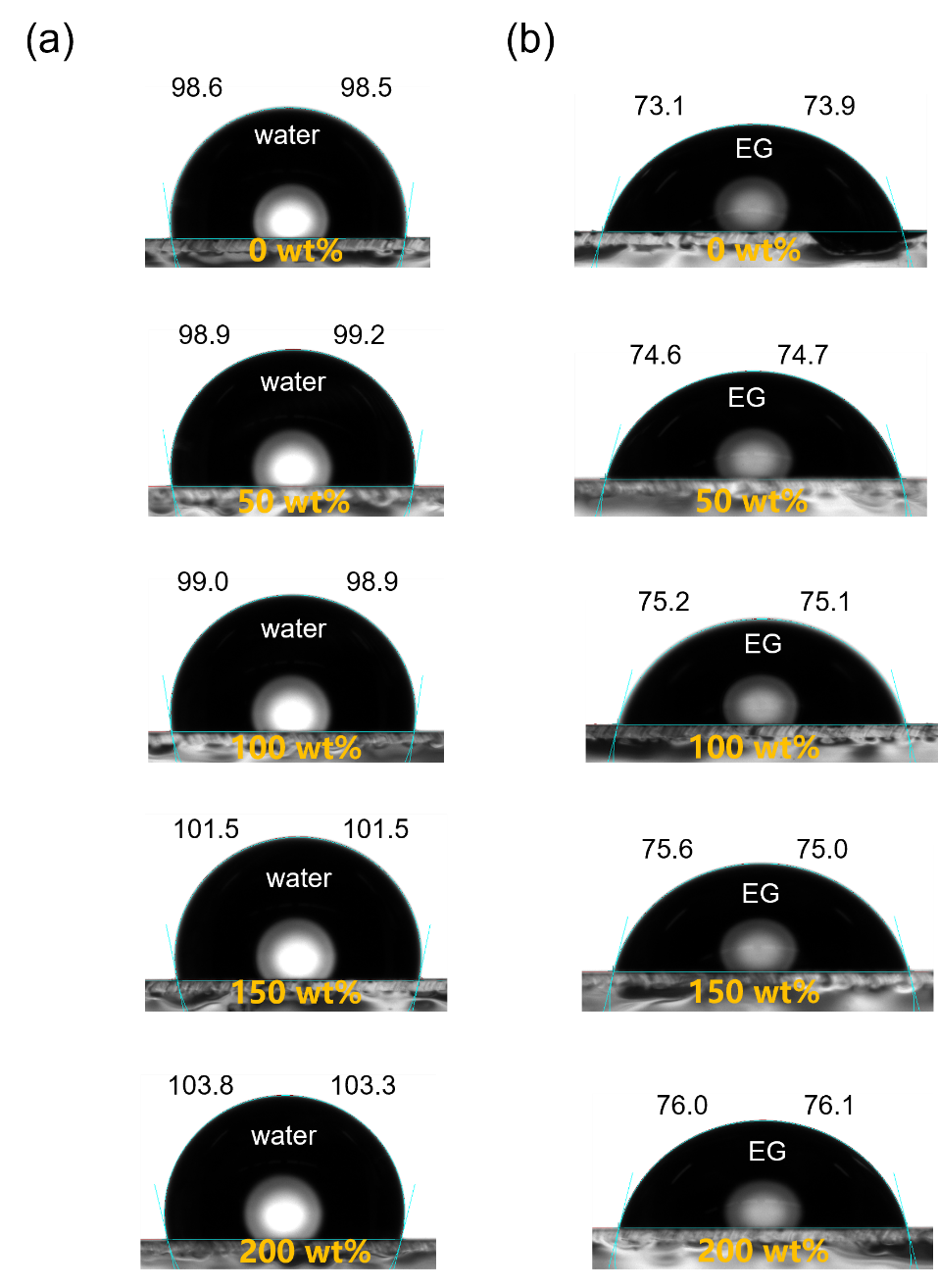
Figure S12.** Contact angles of (a) water, and (b) EG on the water-side SS-PM6:Y6:PC_71_BM:PAT films with different PAT proportion.

**
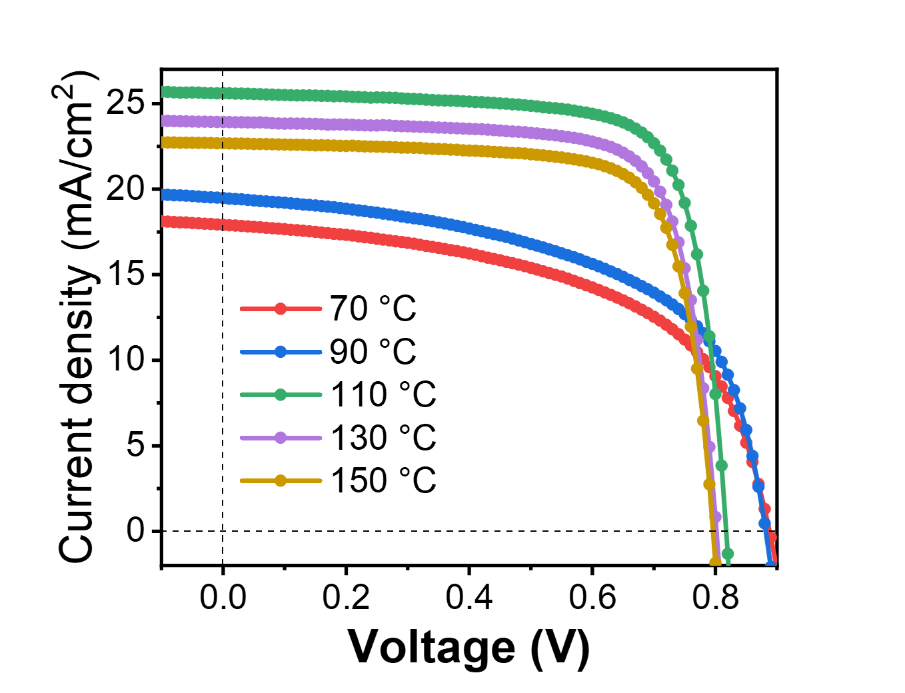
**

# **Figure S13.** *J-V* curves of SS-OPVs with 90 wt% PAT after different annealing temperature at fixed 2 min.

# **Table S1.** Summary of the PCE parameters of aromatic solid additives in OPVs.

| **Additive** | **Active layer** | **Proportion** | **Before PCE** | **After**  **PCE** | **Ref** |
| --- | --- | --- | --- | --- | --- |
| 1,4-diiodobenzene  (DIB) | PM6:Y6 | 172 wt% | 15.39% | 17.36% | ^1^ |
| 1,4-dichlorobenzene (DCB) | PM6:BTP-eC9 | 135 wt% | 16.50% | 17.80% | ^2^ |
| 1-chloro-4-iodobenzene (CIB) | PM6:BTP-eC9 | 135 wt% | 16.50% | 18.40% | ^2^ |
| Anthracene  (An) | PM6:Y6 | 61.4 wt% | 15.60% | 17.02% | ^3^ |
| Phenanthrene  (PAT) | PM6:Y6 | 70 wt% | 12.16% | 19.04% | ^4^ |
| benzo[b]benzo[4,5]thieno[2,3-d]thiophene  (BTBT) | PM6:Y6 | 20 wt% | 10.25% | 16.37% | ^5^ |
| Fluorene  (FL) | PM6:L8-BO | 30 wt% | 16.60% | 18.10% | ^6^ |
| Dibenzothiophene  (DBT) | PM6:L8-BO | 30 wt% | 16.60% | 17.63% | ^6^ |
| dibenzofuran  (DBF) | PM6:L8-BO | 30 wt% | 16.60% | 18.62% | ^6^ |
| 1-amino-o-carborane (CB-NH2) | PM6:BTP-eC9 | 32 wt% | 17.64% | 19.48% | ^7^ |
| Dithienothiophene  (DTT) | PM6:L15 | 76 wt% | 14.39% | 18.03% | ^8^ |
| TT-Cl | PM6:Y6 | 100 wt% | 15.40% | 17.50% | ^9^ |
| 1,3,5-trimethoxybenzene  (TMB) | PBDB-TF:eC9 | 120 wt% | 17.57% | 18.61% | ^10^ |

1. Proportion is the mass ratio of the additive to the acceptor.
2. Before PCE is the device performance before adding the additive.
3. After PCE is the device performance after adding the additive.

# **Table S2.** The contact angle and surface energy of the different solid additive films.

| **Additive** | **Contact angle (°)** | | **Surface energy (mJ/m)** | | |
| --- | --- | --- | --- | --- | --- |
|  | **Water** | **EG** | **γd** | **γp** | **γ** |
| DIB | 64.7/64.7 | 62.9/62.9 | 2.27 | 38.34 | 40.61 |
| DCB | 62.8/62.9 | 60.3/59.2 | 2.90 | 38.63 | 41.53 |
| Nap | 50.4/60.9 | 57.6/57.8 | 2.94 | 40.56 | 43.50 |
| An | 72.7/72.7 | 71.7/71.8 | 1.88 | 31.67 | 33.55 |
| PAT | 97.3/97.3 | 69.7/69.5 | 24.72 | 1.65 | 26.38 |

# **Table S3.** The results of peak position and area of XPS core levels in SS-Y6 and SS-PM6 films without/with PAT.

| **Solvent** | **SS- Y6** | | **SS-Y6:PAT** | |
| --- | --- | --- | --- | --- |
|  | Peak position (eV) | Atomic (a.u.) | Peak position (eV) | Atomic (a.u.) |
| O 1s | 531.1 | 9197 | 531.1 | 15501 |
|  | 532.4 | 65332 | 532.2 | 17478 |
| N 1s | 399.1 | 11629 | 399.0 | 18831 |
|  | 400.5 | 6310 | 400.5 | 10689 |
| S 2p | 164.2 | 9077 | 164.1 | 13187 |
|  | 165.3 | 4384 | 165.3 | 8895 |

| **Solvent** | **SS-PM6** | | **SS-PM6:PAT** | |
| --- | --- | --- | --- | --- |
|  | Peak position (eV) | Atomic (a.u.) | Peak position (eV) | Atomic (a.u.) |
| O 1s | 530.8 | 13715 | 530.8 | 13481 |
|  | 532.0 | 7760 | 532.0 | 8042 |
| S 2p | 163.9 | 31456 | 163.9 | 30282 |
|  | 165.1 | 16056 | 165.1 | 15466 |

# **Table S4.** The photovoltaic parameters of SS-OPVs made by SS-PM6:Y6:PC_71_BM:PAT (1:1.2:0.12:x, w/w/w/w) films with different PAT proportion.

| **PAT** | ***V*_OC_ (V)** | ***J*_SC_ (mA/cm^2^)** | **FF (%)** | **PCE (%)** |
| --- | --- | --- | --- | --- |
| 50 wt% | 0.786±0.001  0.786 | 25.7±0.33  25.9 | 76.2±0.99  77.3 | 15.4±0.37  15.7 |
| 70 wt% | 0.795±0.003  0.792 | 25.3±0.77  25.6 | 75.5±0.94  76.5 | 15.2±0.59  15.5 |
| 90 wt% | 0.811±0.004  0.814 | 25.4±0.24  25.5 | 78.1±0.76  78.9 | 16.1±0.38  16.4 |
| 100 wt% | 0.814±0.002  0.815 | 22.7±0.85  25.3 | 74.8±0.85  75.1 | 13.8±0.52  14.4 |
| 150 wt% | 0.826±0.004  0.830 | 20.9±0.19  20.8 | 75.1±0.97  75.9 | 13.0±0.24  13.1 |
| 200 wt% | 0.840±0.005  0.844 | 18.6±0.44  18.7 | 73.2±0.95  73.8 | 11.4±0.21  11.6 |

*Statistical values obtained from 10 independent devices.

# **Table S5.** The contact angle and surface energy of the water-side SS-PM6:Y6:PC_71_BM:PAT films with different PAT proportion.

| **PAT (wt%)** | **Contact angle (°)** | | **Surface energy (mJ/m)** | | |
| --- | --- | --- | --- | --- | --- |
|  | **Water** | **EG** | **γd** | **γp** | **γ** |
| 0 | 98.6/98.5 | 73.1/73.9 | 20.92 | 2.03 | 22.95 |
| 50 | 98.9/99.2 | 74.6/74.7 | 20.00 | 2.08 | 22.08 |
| 100 | 99.0/98.9 | 75.2/75.1 | 19.22 | 2.27 | 21.49 |
| 150 | 101.5/101.5 | 75.6/75.0 | 22.18 | 1.18 | 23.36 |
| 200 | 103.8/103.3 | 76.0/76.1 | 23.70 | 0.66 | 24.36 |

# **Table S6.** The (010) peaks, corresponding π-π stacking distance (d010), full width at half maximum (FWHM) and crystal coherence length (CCL) of SS-PM6:Y6 and SS-PM6:Y6:PAT films.

| **Film** | **(010) peaks (Å^-1^)** | **d010 (Å)** | | **FWHM (Å^-1^)** | **CCL (Å)** |
| --- | --- | --- | --- | --- | --- |
| w/o PAT | 1.59 | 0.57 | 0.67 | | 3.96 |
| with PAT | 1.68 | 0.55 | 0.65 | | 3.74 |

The peak position and full width at half maximum (FWHM) of the characteristic scattering peak are obtained by peak fitting. The coherent crystallite length (CCL) is calculated by the Scherrer equation (Equation S14).

$CCL=\frac{0.9\times2\pi}{\mathrm{FWHM}}$ (S14)

# **Table S7.** The photovoltaic parameters of SS-OPVs with 90 wt% PAT after different annealing temperature at 2 min.

| **Annealing temperature** | ***V*_OC_ (V)** | ***J*_SC_ (mA/cm^2^)** | | **FF (%)** | **PCE (%)** |
| --- | --- | --- | --- | --- | --- |
| 70 ℃ | 0.885±0.002  0.887 | | 17.9±0.22  17.9 | 56.8±0.76  55.6 | 9.00±0.16  7.81 |
| 90 ℃ | 0.878±0.003  0.881 | | 19.2±0.32  19.4 | 57.4±0.32  57.1 | 9.7±0.21  9.77 |
| 110 ℃ | 0.811±0.004  0.814 | | 25.4±0.24  25.5 | 78.1±0.76  78.9 | 16.1±0.38  16.4 |
| 130 ℃ | 0.797±0.004  0.802 | | 23.3±0.34  23.9 | 75.0±0.80  75.7 | 13.9±0.14  14.5 |
| 150 ℃ | 0.791±0.005  0.796 | | 22.6±0.25  22.7 | 75.5±0.46  75.8 | 13.5±0.29  13.7 |

*Statistical values obtained from 10 independent devices.

# **Table S8.** The photovoltaic parameters of SS-OPVs with PAT after different annealing time at 110 ℃.

| **Annealing time** | ***V*_OC_ (V)** | ***J*_SC_ (mA/cm^2^)** | **FF (%)** | **PCE (%)** |
| --- | --- | --- | --- | --- |
| 0 | 0.869±0.002  0.870 | 21.2±0.4  21.1 | 63.3±1.51  62.1 | 11.7±0.45  11.4 |
| 30 s | 0.850±0.002  0.850 | 22.5±0.38  22.5 | 62.8±0.40  63.1 | 12.0±0.23  12.0 |
| 1 min | 0.818±0.003  0.821 | 25.2±0.43  25.6 | 75.3±0.33  75.5 | 15.5±0.28  15.9 |
| 2 min | 0.811±0.004  0.814 | 25.4±0.24  25.5 | 78.1±0.76  78.9 | 16.1±0.38  16.4 |
| 3 min | 0.798±0.002  0.801 | 25.4±0.36  25.8 | 76.1±0.41  76.6 | 15.4±0.33  15.8 |
| 5 min | 0.797±0.004  0.801 | 25.1±0.35  25.6 | 75.6±1.00  76.4 | 15.1±0.45  15.7 |
| 10 min | 0.786±0.004  0.789 | 24.6±0.39  25.4 | 74.0±0.44  74.3 | 14.3±0.10  14.9 |

*Statistical values obtained from 10 independent devices.

**Reference**

1. Fu, J.; Chen, H.; Huang, P.; Yu, Q.; Tang, H.; Chen, S.; Jung, S.; Sun, K.; Yang, C.; Lu, S., Eutectic phase behavior induced by a simple additive contributes to efficient organic solar cells. *Nano Energy* **2021,** *84*, 105862.

2. Kong, L.; Zhang, Z.; Zhao, N.; Cai, Z.; Zhang, J.; Luo, M.; Wang, X.; Chen, M.; Zhang, W.; Zhang, L., In situ removable additive assisted organic solar cells achieving efficiency over 19% and fill factor exceeding 81%. *Advanced Energy Materials* **2023,** *13* (25), 2300763.

3. Fan, H.; Yang, H.; Wu, Y.; Yildiz, O.; Zhu, X.; Marszalek, T.; Blom, P. W.; Cui, C.; Li, Y., Anthracene‐assisted morphology optimization in photoactive layer for high‐efficiency polymer solar cells. *Advanced Functional Materials* **2021,** *31* (37), 2103944.

4. Fan, H.; Yang, H.; Wu, Y.; Cui, C.; Li, Y., Phenanthrene Treatment for O‐xylene‐Processed PM6: Y6‐Based Organic Solar Cells Enables Over 19% Efficiency. *Advanced Energy Materials*, 2405257.

5. Li, X.; Yang, H.; Fan, H.; Hu, K.; Cao, H.; Cui, C.; Li, Y., Solid additive-assisted morphology optimization enables efficient nonhalogen solvent-processed polymer solar cells. *Journal of Materials Chemistry C* **2023,** *11* (2), 539-545.

6. Xu, J.; Xiao, C.; Zhang, Z.; Zhang, J.; Wang, B.; McNeill, C. R.; Li, W., Utilization of polycyclic aromatic solid additives for morphology and thermal stability enhancement in photoactive layers of organic solar cells. *Small* **2024,** *20* (46), 2405573.

7. Wang, H.; Zhong, Z.; Gámez‐Valenzuela, S.; Lee, J. W.; Li, B.; Xu, C.; Yang, J.; Sun, H.; Kim, B. J.; Liu, B., High‐Performance Organic Solar Cells Enabled by 3D Globally Aromatic Carboranyl Solid Additive. *Advanced Functional Materials* **2025,** *35* (14), 2418805.

8. Liu, B.; Xu, W.; Ma, R.; Lee, J. W.; Dela Peña, T. A.; Yang, W.; Li, B.; Li, M.; Wu, J.; Wang, Y., Isomerized green solid additive engineering for thermally stable and eco‐friendly all‐polymer solar cells with approaching 19% efficiency. *Advanced Materials* **2023,** *35* (49), 2308334.

9. Zhang, H.; Ran, G.; Cui, X.; Liu, Y.; Yin, Z.; Li, D.; Ma, X.; Liu, W.; Lu, H.; Liu, R., Mitigating exciton recombination losses in organic solar cells by engineering nonfullerene molecular crystallization behavior. *Advanced Energy Materials* **2023,** *13* (38), 2302063.

10. Chen, Z.; Yao, H.; Wang, J.; Zhang, J.; Zhang, T.; Li, Z.; Qiao, J.; Xiu, S.; Hao, X.; Hou, J., Restrained energetic disorder for high-efficiency organic solar cells via a solid additive. *Energy & Environmental Science* **2023,** *16* (6), 2637-2645.
